# Catalytic growth of ZnO nanostructures by r.f. magnetron sputtering

**DOI:** 10.1186/1556-276X-6-437

**Published:** 2011-06-24

**Authors:** María Arroyo-Hernández, Raquel Álvaro, Sheila Serrano, José Luis Costa-Krämer

**Affiliations:** 1 IMM-Instituto de Microelectrónica de Madrid (CNM-CSIC), Isaac Newton 8, PTM, Tres Cantos, Madrid 28760, Spain

## Abstract

The catalytic effect of gold seed particles deposited on a substrate prior to zinc oxide (ZnO) thin film growth by magnetron sputtering was investigated. For this purpose, selected ultra thin gold layers, with thicknesses close to the percolation threshold, are deposited by thermal evaporation in ultra high vacuum (UHV) conditions and subsequently annealed to form gold nanodroplets. The ZnO structures are subsequently deposited by r.f. magnetron sputtering in a UHV chamber, and possible morphological differences between the ZnO grown on top of the substrate and on the gold are investigated. The results indicate a moderate catalytic effect for a deposited gold underlayer of 4 nm, quite close to the gold thin film percolation thickness.

## Introduction

Single crystalline zinc oxide (ZnO) nanowires are usually grown by wet chemical and vapour transport methods. The latter are performed at temperatures in the 850 to 1400°C range [1,2]. Lower temperature (400°C) metalorganic vapour-phase epitaxial growth of vertically well-aligned ZnO nanorods has been also reported in [3]. Another kind of nanowires, Si and GaAs, are grown by vapour-liquid-solid deposition (VLS) using gold nanoparticle catalysts [4,5]. Notably, III to V nano-whiskers have been grown on III to V substrates by metalorganic chemical vapour deposition (MOCVD) [6,7]. This approach relies on annealing a thin Au film to form the seed particles [8]. In this way, a homogeneous whisker width distribution is obtained, the mean size of which could be controlled by the thickness of the Au layer and the way this layer transforms to nanoparticles. A similar approach to form ZnO nanostructures is reported herein, but using r.f. magnetron sputtering in ultra high vacuum (UHV) conditions, as a first step towards size-, shape- and position-controlled nanowires, similarly to what Samuelson and coworkers [9] started in GaAs in 2001. Our approach aims at obtaining nanostructures with low level of impurities for future studies on the correlation between defects and transport and photonic properties.

## Experimental

ZnO films were grown on both silicon (100) and sapphire (Al_2_O_3_) substrates by a ZnO target magnetron sputtering. The stoichiometry of the films was checked under different growth conditions by non-RBS spectroscopy. Prior to the ZnO growth, a gold ultra thin underlayer was deposited by thermal deposition at 0.2 Å/s deposition rate. The base pressure is 10^-8 ^mbar and increases slightly to approx. 10^-7 ^mbar during the deposition process. For comparison purposes, a gold pattern was predefined on the substrate. This gold pattern allowed a straightforward comparison of possible ZnO morphology differences on a subsequent scanning electron microscopy inspection. The pattern was defined by electron lithography: a 200-nm PMMA-A4 resin was deposited by spinning for 1 min at 5000 revolutions per minute. Subsequently, they were cured on a hot plate for 4 min at 180°C. For the lithography, a high-resolution LEO 1455 scanning electron microscopy was used. Finally, the developing process was performed by immersing the samples in 4-methyl-2-pentanone + isopropyl alcohol (1:3) for 1.5 min and a subsequent rinse in isopropyl alcohol for 30 s to stop the process. After the development, the patterns were coated with desired gold thickness and subsequently lifted off in acetone.

The gold films were thermally annealed using a tungsten wire heater placed below the holder substrate inside the UHV sputtering system. The annealing was performed for 20 min at 450°C in 10^-2 ^mbar Ar pressure. The ZnO structures were grown by r.f. magnetron sputtering using a ZnO target. The base pressure is 10^-8 ^mbar to ensure a low level of impurities. The growing conditions are: 100 W r.f. power, 500°C, 10^-2 ^mbar Ar pressure, to ensure good crystallographic and conducting properties [10].

The atomic force microscopy (AFM) analysis was performed using a commercial AFM (Nanotec, Madrid Spain) microscope, measuring in contact mode. Commercial tips (Nanosensors, Neuchatel, Switzerland) were used with *K *= 36 to 58 N/m and resonant frequencies in the 328 to 359 KHz range.

The X-ray diffraction (XRD) experiments were performed using a Philips X-PERT four-cycle diffractometer with a Cu Ka radiation source in Bragg-Brentano geometry. The 2theta-omega range scanned was 30° to 95°. The crystal gold grain size was calculated from the diffractogram peak shape using Scherrer equation:(1)

where the shape factor *k *= 0.9, λ is 1.54Å, β the full width half maximum (FWHM) and θ the Bragg angle.

## Results

**Figure 1:**
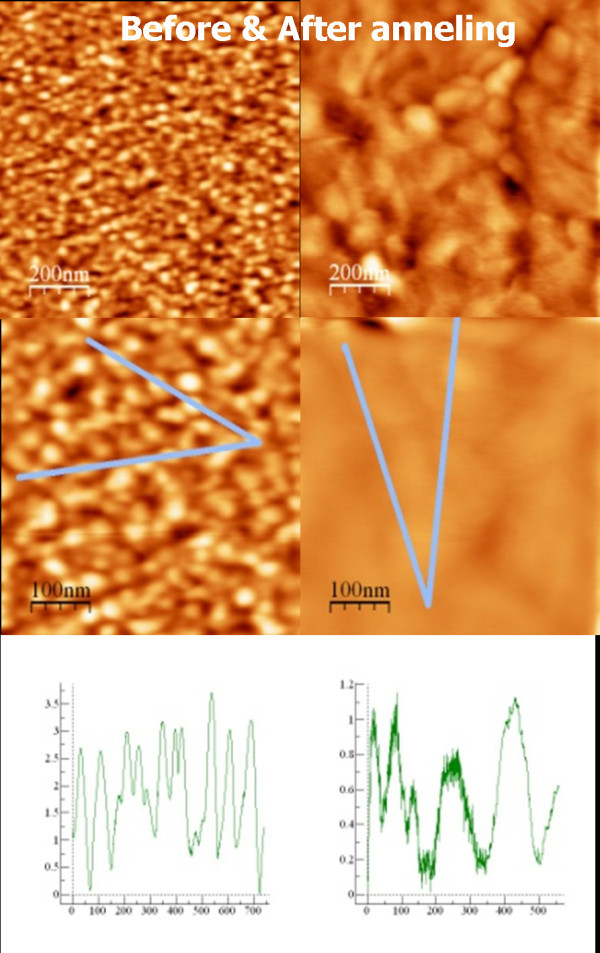
**AFM pictures showing the morphology variations-grain size and roughness-due to thermal annealing for a 3-nm gold thickness film**. Left images are before and right ones are after the annealing.

Our approach to obtain gold seed particles relies on annealing ultra thin gold films. This annealing enhances atomic mobility and produces morphological changes that proceed towards island formation [8] or 3D growth. To check the effect of the thermal annealing on the gold film properties, AFM and XRD were measured for a selected 3-nm thickness gold film. This thickness was chosen because film percolation growth takes place around this nominal thickness value [11]. Figure 1 shows the AFM images obtained before (left pictures) and after (right pictures) the annealing. There are shown two fields of view, corresponding to 200-μm scale bar (top) and to 100-μm scale bar (bottom). The profiles correspond to the lines marked on the pictures. The morphological grain size increases from 27 to 100 nm and the surface roughness decreases from 0.72 to 0.54 nm, which confirms the mobility of gold atoms on the surface.

**Figure 2:**
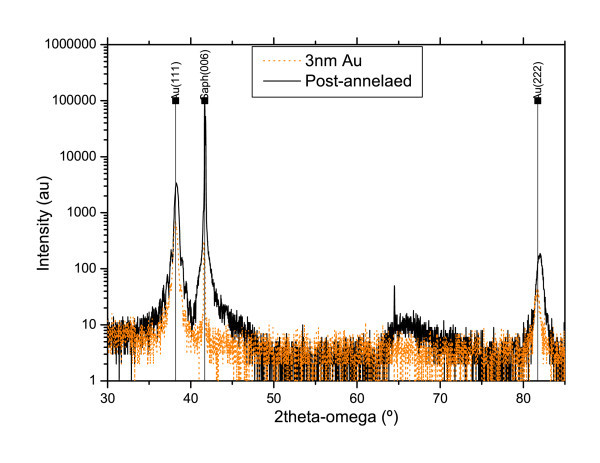
**XRD pattern of a 3-nm nominal thickness gold film before (orange) and after (black) thermal annealing**.

The XRD spectra show that the evaporated gold is textured (111) with a crystallographic grain size of 27.5 nm (calculated from Equation 1), the same as the morphological value obtained by AFM. After the annealing process, the XRD grain size slightly increases up to 28.2 nm, representative of a moderate effect of the thermal treatment in the crystal quality (Figure 2). This marked difference with the AFM results can be understood in terms of 3D changes produced by the atomic mobility.

**Figure 3:**
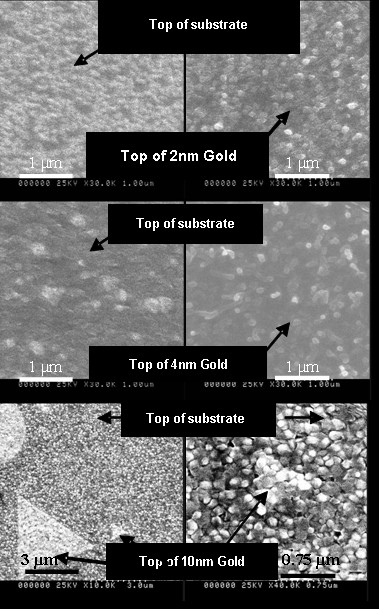
**SEM top views comparing the ZnO structures grown directly onto the substrate and onto an annealed ultra thin gold film of different thicknesses**.

**Figure 4:**
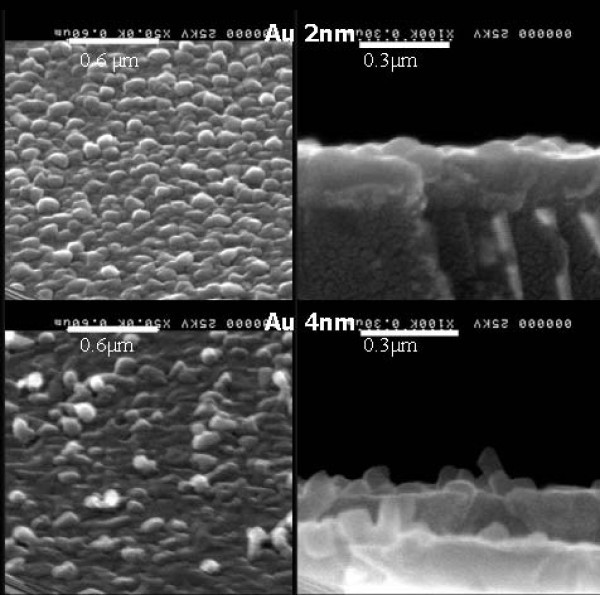
**SEM tilted images and cross-sectional views of ZnO structures grown on 2 and 4 nm gold film nominal thickness**.

To check a possible gold catalytic effect of the ZnO growth on gold, this was carried out for 2, 4 and 10 nm thin gold thicknesses. The morphology of the ZnO structures grown was inspected by SEM (Figure 3). We deliberately went above and below the gold percolation threshold to avoid irreproducible and drastic morphological and temperature dependencies inherent to percolation behaviour. To evaluate this possible catalytic effect of gold, the morphology of ZnO directly growth on sapphire (Figure 3, left) and on top of the annealed gold (Figure 3, right) were compared. As seen, there is no difference between both for 10 nm gold films. On the other hand, ZnO grown on 2 nm gold thickness shows a different structure depending on the material below, but only for 4 nm gold films marked differences are observed. In Figure 4, detailed images illustrating this effect are shown. The cross-sectional views clearly show a randomly oriented ZnO nanostructure when grown on 4 nm gold films, with lengths ranging from 80 to 220 nm. This confirms the moderate effect of gold for catalytic ZnO nanostructure formation.

**Figure 5:**
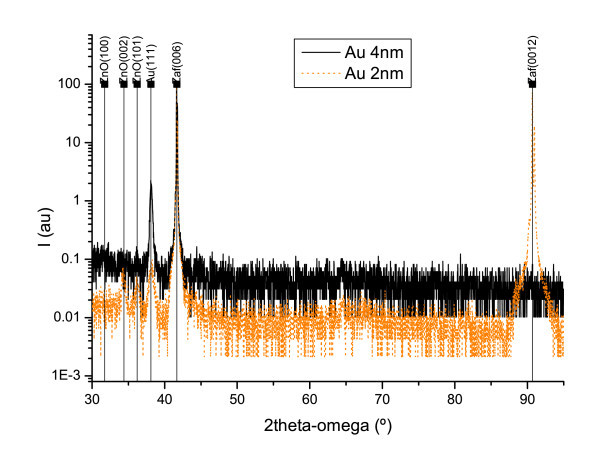
**XRD diffraction spectra of ZnO structures grown on 2 nm (orange) and 4 nm (black) gold film nominal thicknesses**.

Finally, XRD was studied for both kinds of ZnO structures (Figure 5). The spectra show the diffraction peaks associated to the sapphire substrate and to the gold film. It can be observed that ZnO grown on 2-nm thickness gold has a polycrystalline structure, with two preferential orientations: (002) and (101). On the other hand, the ZnO structures grown on the 4-nm thickness gold are 'XRD amorphous'.

## Conclusions

In summary, experiments addressing a possible catalytic effect of gold on ZnO growth by r.f. magnetron sputtering under UHV conditions are presented. A moderate catalytic effect of gold is reported. The maximum effect is measured to happen at intermediate ultra thin gold nominal thicknesses, around 4 nm, and a subsequent thermal annealing at 450°C. This nominal thickness is slightly larger than the gold percolation one. The obtained ZnO nanostructures show a random orientation and are XRD amorphous. At this thickness range, the effect of the substrate temperature, the nominal ZnO thickness and the partial pressure composition during ZnO growth could be used to improve the catalytic effect and the nanostructure quality.

## Abbreviations

AFM: atomic force microscopy; FWHM: full width half maximum; MOCVD: metalorganic chemical vapour deposition; UHV: ultra high vacuum; XRD: X-ray diffraction; ZnO: zinc oxide.

## Competing interests

The authors declare that they have no competing interests.

## Authors' contributions

MA-H, with the help of JLC-K and SG perform the UHV magnetrón growth of the ZnO, MA-H, and JLCK performed the ultrathin Au metal growth, RA carried out the mask design, spinning and e-beam lithography, MA-H, performed the X-ray experiments, JLC-K with MA-H performed the SEM film preparation and imaging experiments. AFM experiments were performed by MA-H. JLC-K and MA-H conceived the study, and participated in its design and coordination
